# White matter hyperintensity drives EEG microstate abnormalities in arteriosclerotic cerebral small vessel disease

**DOI:** 10.3389/fnins.2026.1865573

**Published:** 2026-07-31

**Authors:** Kaiyan Feng, Jiaxin Cai, Jianming Lei, Guofang Zeng, Hai Chen, Jiaxin Chen, Qishan He, Lijuan Liang, Yizhong Li, Xiong Zhang, Hao Li, Shisheng Ye

**Affiliations:** 1 Department of Neurology, Maoming People’s Hospital, Maoming, China; 2 The First School of Clinical Medicine, Southern Medical University, Guangzhou, China; 3 Department of Integrated Therapy, Maoming People’s Hospital, Maoming, China; 4 The First School of Clinical College of Medicine, Guangdong Medical University, Zhanjiang, China; 5 Department of Neurology, Dongguan Chang’an Hospital, Dongguang, China; 6 Department of Neurology, Leliu Hospital Affiliated with Shunde Hospital, Guangzhou University of Chinese Medicine, Foshan, China; 7 Department of Radiology, Maoming People’s Hospital, Maoming, China

**Keywords:** arteriosclerotic cerebral small vessel disease, dementia, electroencephalogram, microstate analysis, white matter hyperintensity

## Abstract

**Objectives:**

Although MRI is the optimal imaging method for assessing white matter hyperintensity (WMH), it lacks sensitivity to the underlying pathophysiological changes of WMH. This study aims to investigate EEG changes associated with WMH and identify potential EEG biomarkers for WMH-related dementia.

**Methods:**

This cross-sectional study enrolled 90 subjects: 30 patients with WMH with dementia (WMHD), 30 patients with WMH without dementia (WMH-ND), and 30 age-and sex-matched healthy controls. Brain MRI was used to segment and quantify deep, periventricular, and total WMH volumes (DWMH, PVWMH, TWMH). Resting-state EEG data were recorded for microstate analysis. Four microstate classes (A, B, C, D) were extracted, and their occurrence, mean duration, time coverage, and transition probabilities were calculated. Partial correlation analysis was used to evaluate the associations between microstate features and WMH volume as well as MMSE scores. Based on microstate features, classification prediction models were constructed using machine learning algorithm.

**Results:**

In the WMHD group, the temporal parameters (occurrence, mean duration, time coverage) of microstate C were decreased (*P* < 0.05–0.001), while the mean duration of microstates A and B was prolonged (*P* < 0.05–0.001). The WMH-ND group also showed reduced microstate C temporal parameters (*P* < 0.001), but exhibited compensatory increases in mean duration and time coverage of microstate D (*P* < 0.001). Transition probabilities toward microstate C were reduced in both patient groups (*P* < 0.001), and all transition probabilities toward microstate D were lower in WMHD than in WMH-ND (*P* < 0.05–0.001). Temporal parameters of microstates A and B and transition probabilities toward them were positively correlated with WMH volume and negatively correlated with MMSE score. Conversely, those of microstate D showed the opposite correlations. For distinguishing WMHD from WMH-ND, support vector machine (SVM) and logistic regression (LR) performed best (accuracy: 76.67%, AUC: 0.84). Classification performance was highest for WMH-ND vs. HC (LR: 79.17%, AUC 0.86), and lowest for WMHD vs. HC (SVM: 68.33%, AUC 0.77).

**Conclusion:**

Patients with WMH exhibit alterations in EEG microstate features that are significantly correlated with WMH burden and cognitive function. Classification models based on these microstate features show promise for practical application in WMH-related dementia.

## 1 Introduction

Arteriosclerotic cerebral small vessel disease (aCSVD) is the most common type of cerebral small vessel disease (CSVD) (Litak et al., 2020). White matter hyperintensity (WMH) is the most common MRI manifestation of aCSVD (Duering et al., 2023). Approximately 50% of people develop WMH by age 50 (Wen et al., 2009), and up to 95% by age 90 (de Leeuw et al., 2001). Clinically, WMH is significantly associated with cognitive decline and dementia (Huang et al., 2024). However, the relationship between WMH and cognitive function is complex; for example, WMH volume, spatial location, and shape are all associated with cognitive impairment (Zhang et al., 2023; Qiao et al., 2022; Karvelas and Elahi, 2023). Even among patients with similar WMH burden, cognitive function can vary considerably, suggesting that conventional imaging assessments lack sensitivity to the underlying pathophysiological changes of WMH (de Leeuw et al., 2001).

The application of electroencephalography (EEG) microstate analysis in the study of cognitive dysfunction has gained increasing attention. By capturing the transient spatial topographical patterns of EEG signals, microstates can reflect the dynamic reorganization of functional brain networks, offering a perspective on the neural mechanisms underlying cognitive impairment (Lehmann et al., 1987; Michel and Koenig, 2018). Previous studies have shown that microstate features are related to cognitive function and may serve as potential biomarkers for early identification of cognitive impairment (Lassi et al., 2023; Shi et al., 2022; Wu et al., 2025; Engedal et al., 2020). In the context of CSVD, emerging evidence also suggests atypical EEG microstate dynamics. For instance, CSVD patients with cognitive impairment exhibited significantly lower occurrence rates for all four canonical microstates (A, B, C, and D) compared to healthy controls, alongside prolonged durations of microstates B, C, and D, and a reduced transition probability from microstate C to A. Furthermore, the occurrence rate of microstate D was positively correlated with MMSE scores, whereas the transition from A to B was negatively correlated with cognitive performance, indicating the potential of microstate metrics to track disease severity in CSVD (Jin et al., 2025). Therefore, this study aims to investigate EEG changes associated with WMH and identify potential EEG biomarkers for WMH-related dementia.

## 2 Materials and methods

This was a cross-sectional study. The study protocol was approved by the Ethics Committee of Maoming People’s Hospital (PJ2025MI-K021-01). All participants gave written informed consent; for those unable to provide consent, a direct relative signed on their behalf.

### 2.1 Participants

This was a cross-sectional study. From October 2022 to April 2025, we consecutively screened and enrolled 60 patients with aCSVD and severe WMH (Fazekas score 3) from the Department of Neurology, Maoming People’s Hospital. Based on Mini-Mental State Examination (MMSE) score, they were divided into two groups: WMH without dementia group (WMH-ND, *n* = 30) and WMH with dementia group (WMHD, *n* = 30). In addition, 30 age-and sex-matched healthy controls (HC) were recruited from the physical examination center.

### 2.2 Diagnostic criteria for aCSVD

Diagnostic criteria for aCSVD (Ye et al., 2025): All of the following conditions must be met: (1) Presence of at least one atherosclerotic risk factor: smoking (≥ 10 cigarettes/day and not abstinent), body mass index (BMI) > 28 kg/m^2^, hypertension, diabetes, impaired fasting glucose or impaired glucose tolerance, hyperlipidemia, hyperhomocysteinemia, or atherosclerotic cardiovascular disease; (2) Presence of at least one CSVD-related clinical symptom, including but not limited to: transient ischemic attack, acute lacunar syndrome, cognitive decline, gait or postural balance disturbance, vascular parkinsonism, sleep disturbance, mood disorder, or urinary/bowel dysfunction; (3) Presence of at least one of the following neuroimaging features on MRI: ≥ 1 lacunar infarct; Fazekas score ≥ 1 for periventricular WMH (PVWMH) and/or deep WMH (DWMH); or ≥ 1 cerebral microbleed.

### 2.3 Inclusion and exclusion criteria

Inclusion criteria for the WMH-ND and WMHD groups: (1) Age ≥ 60 years; (2) Presence of aCSVD-related WMH; (3) WMH meeting the neuroimaging criteria of the STRIVE-1 international standard for CSVD (Duering et al., 2023); (4) Fazekas score of 3 for WMH; (5) The WMH-ND group was defined by an MMSE score indicating no dementia, while the WMHD group was defined by an MMSE score indicating dementia. To account for educational level, the following MMSE cut-offs were used (Li et al., 2016): Education ≤ 0 years (illiterate): MMSE ≤ 17 for dementia; Education 1–6 years: MMSE ≤ 19 for dementia; Education ≥ 7 years: MMSE ≤ 24 for dementia.

Exclusion criteria: (1) WMH caused by cerebral amyloid angiopathy, hereditary cerebral small vessel disease, or inflammatory/immune-mediated cerebral small vessel disease; (2) Significant structural lesions on MRI: > 3 lacunar infarcts, > 5 microbleeds, > 10 enlarged perivascular spaces, or marked cortical or hippocampal atrophy; (3) Extracranial or intracranial arterial stenosis causing hemodynamic changes (lumen stenosis > 50%); (4) Cognitive impairment due to other neurodegenerative disorders, such as Alzheimer’s disease, Parkinson’s disease, frontotemporal dementia, or Lewy body dementia; (5) History of severe cerebrovascular events: large artery atherosclerotic stroke, cardioembolic stroke, intracerebral hemorrhage, or subarachnoid hemorrhage; (6) Concurrent diseases that may affect central nervous system function, including but not limited to: infectious diseases (e.g., encephalitis, meningitis), metabolic diseases (e.g., hepatic encephalopathy, uremic encephalopathy), toxic disorders, primary or metastatic intracranial tumors, and traumatic brain injury; (7) Severe underlying systemic diseases: active pulmonary infection, decompensated heart failure (NYHA grade ≥ II), severe renal impairment (eGFR < 30 ml/min/1.73 m^2^), or hepatic insufficiency (alanine aminotransferase/aspartate aminotransferase > 3 times the upper limit of normal, or total bilirubin > 3 times the upper limit of normal); (8) Conditions interfering with MMSE assessment: psychiatric disorders, severe visual or hearing impairment, aphasia, or dysarthria.

### 2.4 WMH segmentation and quantification protocol

The protocol for WMH segmentation and quantification was as follows. First, FLAIR and T1-weighted images were registered using the LST (Lesion Segmentation Tool, v3.0.0)^1^ toolbox within SPM12 (Statistical Parametric Mapping, v12) running on MATLAB (v9.9). After registration, the total WMH (TWMH) volume was automatically segmented from the co-registered FLAIR images using the lesion prediction algorithm (LPA) (Schmidt, 2017). Subsequently, the lateral ventricles and PVWMH were segmented using anatomical tools from FSL (FMRIB Software Library, v6.0) (Kempton et al., 2011) to generate lateral ventricle masks. PVWMH was defined as lesions within ≤ 10 mm of the lateral ventricles, while DWMH was defined as subcortical lesions located > 10 mm from the ventricles (Griffanti et al., 2017). DWMH volume was calculated by subtracting the PVWMH volume from the total WMH volume. In this study, TWMH was calculated as the sum of DWMH and PVWMH (TWMH = DWMH + PVWMH), with no additional subcortical or juxtacortical WMH subcategories included. Two trained researchers independently assessed the segmentation quality of all images by visual inspection. The automatically generated WMH masks were compared with the original FLAIR images, and each slice was classified into one of three categories: accurate segmentation, over-segmentation, or under-segmentation. The two researchers were blinded to each other’s assessments. In cases of disagreement, a third senior researcher reviewed the images and made the final judgment.

### 2.5 EEG data preprocessing and microstate analysis

EEG signals were recorded using a Nihon Kohden EEG-1200C video EEG system, with 19 Ag/AgCl disc recording electrodes placed strictly according to the international 10-20 system and bilateral mastoid reference electrodes. The resting EEG was recorded for 20 min with the participant’s eyes closed, at a sampling rate of 1,000 Hz. EEG data processing was performed using the EEGLAB toolbox (v2023.0) under MATLAB 2023b (MathWorks Inc., United States). The preprocessing procedure was as follows. First, electrode localization was performed. Bad channels were then repaired using spherical spline interpolation, followed by data screening via visual inspection to exclude segments with amplitude drift exceeding ± 200 μV. The data were subsequently downsampled to 100 Hz. Whole-brain average reference was applied for re-referencing. For frequency filtering, the raw EEG data were band-pass filtered using a finite impulse response filter with a passband of 2–20 Hz. Independent component analysis (ICA) (Anemüller et al., 2003) was used to correct artifacts caused by eye movements, blinks, and muscle activity. After artifact correction, data quality control was conducted, during which artifactual components were re-identified, and segments with large residual artifacts were removed. Finally, for each subject, two high-quality segments of 180 s each were extracted as experimental data (Li et al., 2024), yielding a total of 180 EEG segments (60 each from the WMHD, WMH-ND, and HC groups).

Microstate analysis was performed separately for the WMHD, WMH-ND, and HC groups using the Microstate 1.0 toolbox (Poulsen et al., 2018). For each group, up to 1,000 topographies at local global field power (GFP) maxima (minimum inter-peak distance 100 ms) were extracted per participant and polarity-normalized by inverting any map whose spatial correlation with the within-group mean topography was negative. The normalized topographies from all subjects in a group were concatenated and submitted to modified k-means clustering (100 random initializations) with k ranging from 2 to 9; the optimal cluster number was determined by the cross-validation (CV) index and the global explained variance (GEV) curve, both of which consistently yielded an optimum at *k* = 4 across all groups, and the resulting four group-specific templates matched the canonical microstate classes A-D (Koenig et al., 2002). Spatial correlation coefficients between our group-specific templates and the canonical Koenig templates ranged from 0.75 to 0.84 across all microstate classes and groups. These templates were then back-fitted to each subject’s continuous EEG by assigning every time point to the template with the highest absolute spatial correlation (polarity ignored), and the label sequences were temporally smoothed using a segment-rejection algorithm that discarded intervals shorter than 30 ms. From the smoothed sequences, standard microstate parameters—mean duration, occurrence, time coverage, and transition probabilities—were extracted for subsequent statistical comparisons.

### 2.6 Classification prediction models

This study employed machine learning approaches to construct classification models for distinguishing WMHD from WMH-ND, WMH-ND from HC, and WMHD from HC. First, all features were normalized using min-max scaling to eliminate scale differences. Feature selection was performed using support vector machine-based recursive feature elimination (SVM-RFE) combined with collinearity filtering (Wang et al., 2019); the entire procedure was conducted within a leave-one-out cross-validation (LOOCV) framework to ensure unbiased evaluation. Four algorithms were compared for model construction: support vector machine (SVM) with a linear kernel and parameter tuning (Keerthi and Lin, 2003), Gaussian naïve Bayes (GNB) based on probabilistic modeling (Domingos and Pazzani, 1997), logistic regression (LR) with L2 regularization (Richardson et al., 2018), and neural network (NN) employing a multilayer perceptron architecture (Bourquin et al., 1997). All models were optimized and evaluated via stratified LOOCV with an inner 5-fold cross-validation for hyperparameter tuning. Model performance was assessed using accuracy, precision, recall, F1-score, and receiver operating characteristic (ROC) curves.

### 2.7 Statistical analysis

All statistical analyses and visualizations in this study were performed using MATLAB 2023b. Continuous data are presented as mean ± standard deviation (SD). For comparisons of EEG features among the three groups: if data from all groups followed a normal distribution, one-way analysis of variance (ANOVA) was used to test for overall intergroup differences. If the ANOVA result was statistically significant (*P* < 0.05), Tukey’s honest significant difference (HSD) test was further used for pairwise comparisons. If EEG feature data from any group did not follow a normal distribution, the Kruskal-Wallis H test was used for overall intergroup differences. If the Kruskal-Wallis test result was significant (*P* < 0.05), the Mann-Whitney U test was used for pairwise comparisons, and the Bonferroni correction was applied to control for type I error inflation due to multiple comparisons.

To explore the relationships between EEG features, WMH volume, and MMSE score, partial correlation analyses were performed with age and sex as control variables. Given that multiple variable relationships were analyzed, the Benjamini–Hochberg method was applied to adjust the raw *P*-values from all partial correlation analyses for false discovery rate (FDR). For all statistical analyses, an adjusted *P* < 0.05 was considered statistically significant.

## 3 Results

### 3.1 Comparison of baseline characteristics among groups

The WMHD, WMH-ND, and HC groups were comparable in demographic characteristics. No significant differences were observed among the three groups in age (WMHD: 70.4 ± 5.6 years, WMH-ND: 69.1 ± 5.6 years, HC: 67.3 ± 5.1 years), sex distribution (male proportion: 56.7% vs. 66.7% vs. 60.0%), or years of education (the predominant category in all three groups was 1–6 years of education, accounting for 46.7, 50.0, and 40.0%, respectively; all *P* > 0.05). Regarding MMSE score, WMHD group was significantly lower than HC group (16.0 vs. 27.5, *P* < 0.001) and WMH-ND group (16.0 vs. 24.5, *P* < 0.001). In terms of WMH volume, DWMH (5.3 ml vs. 6.4 ml), PVWMH (21.4 mL vs. 18.2 mL), and TWMH (27.4 mL vs. 27.4 mL) in both the WMHD and WMH-ND groups were significantly higher than HC group (0.1, 1.1, and 1.3 mL, respectively; all *P* < 0.001). Between the WMHD and WMH-ND groups, only MMSE score showed a statistically significant difference (Table 1).

**TABLE 1:** Comparison of baseline characteristics among groups.

Variables	WMHD (*n* = 30)	WMH-ND (*n* = 30)	HC (*n* = 30)
Age (year)	70.4 ± 5.6	69.1 ± 5.6	67.3 ± 5.1
Male, *n* (%)	17 (56.7%)	20 (66.7%)	18 (60.0%)
Education year, n (%)			
0 year	6 (20.0%)	3 (10.0%)	4 (13.3%)
1–6 years	14 (46.7%)	15 (50.0%)	12 (40.0%)
≥7 years	10 (33.3%)	12 (40.0%)	14 (46.7%)
MMSE	16.0 (12.0, 20.0)***^ ###^	24.5 (21.8, 26.0)**	27.5 (27.0, 28.3)
DWMH (ml)	5.3 (4.0, 8.5)***	6.4 (3.4, 11.1)***	0.1 (0.0, 0.3)
PVWMH (ml)	21.4 (15.2, 34.8)***	18.2 (13.6, 27.6)***	1.1 (0.5, 2.6)
TWMH (ml)	27.4 (19.7, 42.7)***	27.4 (18.0, 40.2)***	1.3 (0.5, 3.0)

WMHD vs. HC: *P < 0.05,

**P < 0.01,

***P < 0.001; WMH-ND vs. HC:

*P < 0.05, **P < 0.01, ***P < 0.001; WMHD vs. WMH-ND: ^#^*P* < 0.05, ^##^*P* < 0.01,

^###^*P* < 0.001.

Regarding segmentation quality control, a total of 1,835 image slices were visually evaluated across all participants. Among these, 1,770 slices (96.46%) were judged as accurately segmented, 65 slices (3.54%) showed over-segmentation, and 2 slices (0.11%) showed under-segmentation. The overall agreement between the automated segmentation and the visual inspection-based gold standard, as measured by the Dice coefficient, was 0.98.

### 3.2 Comparison of microstate topographies among groups

**FIGURE 1:**
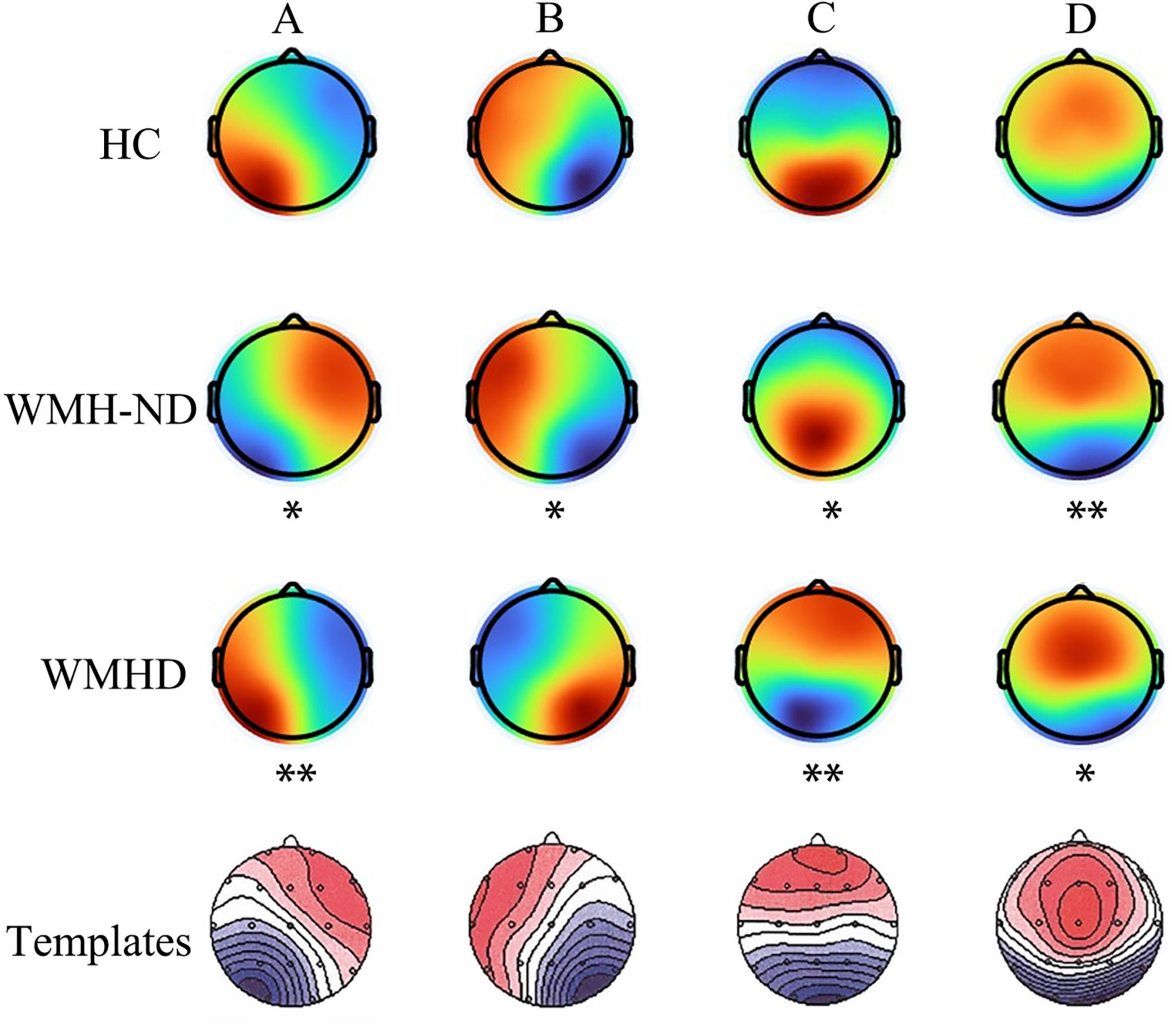
EEG microstate topographies in WMHD, WMH-ND, HC groups and templates (Koenig et al., 2002). Labels **(A–D)** corresponds to microstates A, B, C, and D, respectively. The color scale represents normalized voltage amplitudes, with red indicating positive values and blue indicating negative values (polarity is reversible). Results of TANOVA comparing WMH-ND group and WMHD group versus HC group: *P < 0.05, **P < 0.01. Voltage amplitudes have been normalized.

Based on the classical four-microstate model (Koenig et al., 2002), four microstate classes (A, B, C, D) were identified (Figure 1). Topographic analysis of variance (TANOVA) was performed using Ragu software (Koenig et al., 2011). The results showed topographic differences between the WMH-ND and HC groups for microstates A, B, C, and D (*P* = 0.037; *P* = 0.044; *P* = 0.045; *P* = 0.002, respectively). Topographic differences were also observed between the WMHD and HC groups for microstates A, C, and D (*P* = 0.002; *P* = 0.001; *P* = 0.026, respectively). However, no significant topographic differences were found between the WMHD and WMH-ND groups. The global explained variance (GEV) values for the three groups were 71.20% (WMHD), 72.96% (WMH-ND), and 70.82% (HC), with no significant differences among the groups (all *P* > 0.05).

### 3.3 Comparison of microstate temporal parameters among groups

**FIGURE 2:**
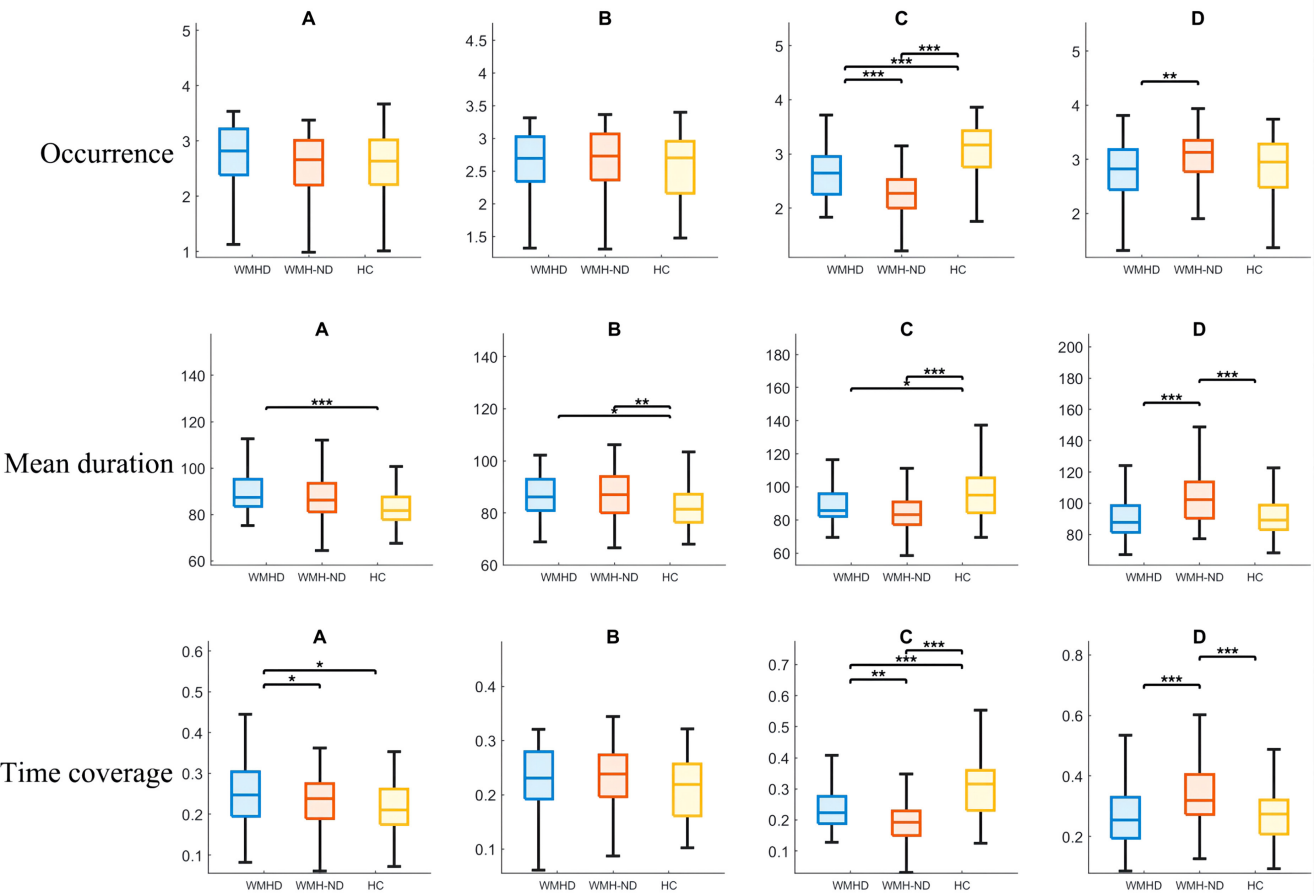
Comparison of microstate temporal parameters (occurrence, mean duration, and time coverage) among WMHD, WMH-ND, and HC groups. Labels **(A–D)** correspond to microstates A, B, C, and D, respectively. *P < 0.05, **P < 0.01, ***P < 0.001.

Comparisons of temporal parameters among the three groups were summarized in Table 2 and Figure 2. Regarding occurrence, the occurrence of microstate C in the WMHD group (2.66 ± 0.48/s) was lower than in HC group (3.03 ± 0.53/s, *P* < 0.001) but higher than in WMH-ND (2.22 ± 0.48/s, *P* < 0.001); WMH-ND also showed lower occurrence of microstate C than HC (*P* < 0.001). The occurrence of microstate D was lower in the WMHD (2.73 ± 0.60/s) than in WMH-ND (3.06 ± 0.51/s, *P* < 0.05). Regarding mean duration, in the WMHD group, mean durations of microstate A and microstate B were longer than in HC (microstate A: 90.44 ± 10.75 ms vs. 82.80 ± 8.06 ms, *P* < 0.001; microstate B: 85.96 ± 8.07 ms vs. 81.94 ± 7.69 ms, *P* < 0.05), whereas microstate C was shorter than in HC group (89.84 ± 12.16 ms vs. 96.16 ± 15.85 ms, *P* < 0.05). Microstate D duration was shorter in WMHD than in WMH-ND (91.10 ± 15.04 ms vs. 106.45 ± 20.64 ms, *P* < 0.001). In the WMH-ND group, microstate C duration was shorter than in HC (84.66 ± 12.29 ms vs. 96.16 ± 15.85 ms, *P* < 0.001), while microstate D duration was longer than in HC (106.45 ± 20.64 ms vs. 90.99 ± 12.30 ms, *P* < 0.001). Regarding time coverage, microstate A coverage in WMHD (0.26 ± 0.08) was higher than in WMH-ND (0.22 ± 0.07) and HC (0.22 ± 0.06) (both *P* < 0.05). Microstate C coverage in WMHD (0.24 ± 0.08) was higher than in WMH-ND (0.19 ± 0.07, *P* < 0.01) but lower than in HC (0.30 ± 0.09, *P* < 0.001); WMH-ND also had lower coverage than HC (*P* < 0.001). Microstate D coverage in WMH-ND (0.34 ± 0.12) was higher than in WMHD (0.26 ± 0.10, *P* < 0.001) and HC (0.27 ± 0.08, *P* < 0.001).

**TABLE 2:** Comparison of microstate temporal parameters among groups.

Temporal parameters	Microstate classes	WMHD (*n* = 30)	WMH-ND (*n* = 30)	HC (*n* = 30)
Occurrence (/s)	A	2.74 ± 0.54	2.52 ± 0.64	2.59 ± 0.53
	B	2.63 ± 0.51	2.62 ± 0.55	2.56 ± 0.51
	C	2.66 ± 0.48	2.22 ± 0.48	3.03 ± 0.53
	D	2.73 ± 0.60	3.06 ± 0.51	2.87 ± 0.53
Mean duration (ms)	A	90.44 ± 10.75	86.94 ± 10.65	82.80 ± 8.06
	B	85.96 ± 8.07	86.81 ± 10.23	81.94 ± 7.69
	C	89.84 ± 12.16	84.66 ± 12.29	96.16 ± 15.85
	D	91.10 ± 15.04	106.45 ± 20.64	90.99 ± 12.30
Time coverage	A	0.26 ± 0.08	0.22 ± 0.07	0.22 ± 0.06
	B	0.23 ± 0.06	0.23 ± 0.07	0.21 ± 0.06
	C	0.24 ± 0.08	0.19 ± 0.07	0.30 ± 0.09
	D	0.26 ± 0.10	0.34 ± 0.12	0.27 ± 0.08

### 3.4 Comparison of microstate transition probabilities among groups

**FIGURE 3:**
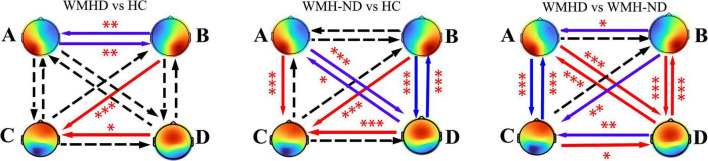
Comparison of microstate transition probabilities among groups. Labels **(A–D)** correspond to microstates A, B, C, and D, respectively. Dashed lines indicate no significant difference between groups. Solid blue lines indicate that WMHD or WMH-ND group is significantly higher than the HC group, or WMHD group is significantly higher than WMH-ND group. Solid red lines indicate that WMHD or WMH-ND group is significantly lower than HC group, or WMHD group is significantly lower than WMH-ND group. *P < 0.05, **P < 0.01, ***P < 0.001.

Comparison of microstate transition probabilities among the three groups (Table 3 and Figure 3) revealed significant differences in multiple transition pathways. Compared with HC group, WMHD group showed increased transition probabilities for A→B and B→A (*P* < 0.01) and decreased probabilities for B→C and D→C (*P* < 0.001 and *P* < 0.05, respectively). Compared with HC, WMH-ND group exhibited increased transition probabilities for A→D, D→A, B→D, and D→B (*P* < 0.05–0.001) and decreased probabilities for A→C, B→C, and D→C (all *P* < 0.001). Furthermore, extensive differences were also observed between the WMHD and WMH-ND groups: the WMHD group had higher transition probabilities for A→C, B→A, B→C, C→A, and D→C (*P* < 0.05–0.001), but lower probabilities for A→D, B→D, C→D, D→A, and D→B (*P* < 0.05–0.001).

**TABLE 3:** Comparison of microstate transition probabilities among groups.

Transition pathways	WMHD (*n* = 30)	WMH-ND (*n* = 30)	HC (*n* = 30)
A→B	0.34 ± 0.08	0.32 ± 0.09	0.29 ± 0.07
A→C	0.33 ± 0.09	0.26 ± 0.07	0.37 ± 0.09
A→D	0.33 ± 0.10	0.42 ± 0.10	0.34 ± 0.09
B→A	0.35 ± 0.10	0.31 ± 0.10	0.29 ± 0.07
B→C	0.30 ± 0.08	0.25 ± 0.07	0.37 ± 0.10
B→D	0.34 ± 0.10	0.44 ± 0.12	0.33 ± 0.09
C→A	0.34 ± 0.08	0.28 ± 0.07	0.31 ± 0.07
C→B	0.30 ± 0.08	0.30 ± 0.07	0.30 ± 0.08
C→D	0.36 ± 0.12	0.42 ± 0.13	0.38 ± 0.09
D→A	0.33 ± 0.08	0.34 ± 0.07	0.30 ± 0.09
D→B	0.32 ± 0.07	0.37 ± 0.08	0.30 ± 0.07
D→C	0.35 ± 0.11	0.28 ± 0.09	0.40 ± 0.11

### 3.5 Microstate correlation with WMH volume and MMSE score

**FIGURE 4:**
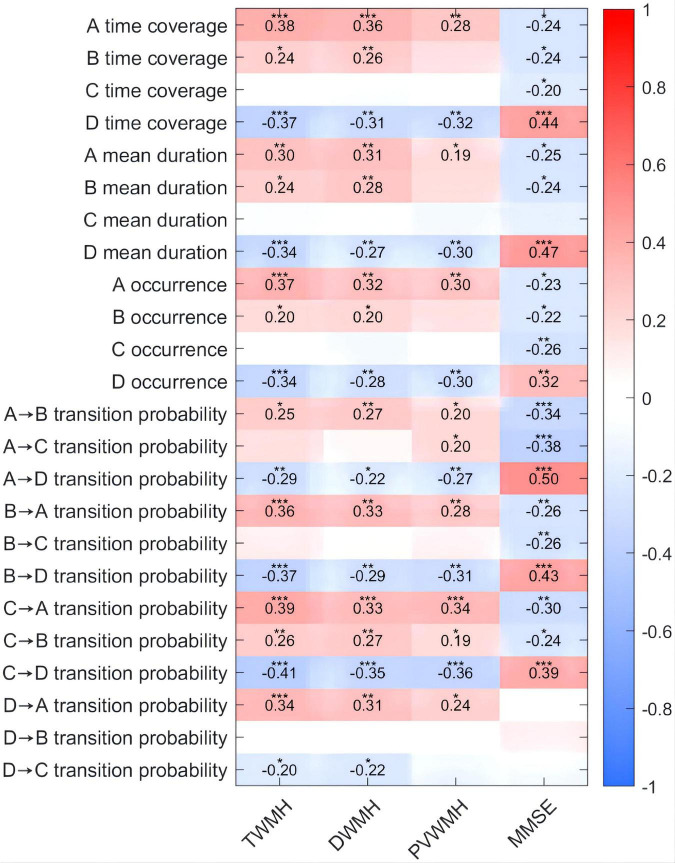
Heatmap of correlation analysis between microstate parameters (temporal parameters and transition probabilities) and WMH volume/MMSE score. Values represent partial correlation coefficients (r) after controlling for age and sex. *FDR < 0.05, **FDR < 0.01, ***FDR < 0.001.

Correlation analysis revealed multiple significant associations between microstate parameters and both WMH volume and MMSE score (Figure 4). Specifically, temporal parameters of microstates A and B were positively correlated with WMH volumes, and negatively correlated with MMSE score. Conversely, temporal parameters of microstate D were negatively correlated with WMH volume and positively correlated with MMSE score. For microstate C, temporal parameters showed no significant correlation with WMH volume; however, its occurrence and time coverage were negatively correlated with MMSE score. Regarding transition probabilities, most pathways involving transitions to microstates A or B were positively correlated with WMH volume and negatively correlated with MMSE score. In contrast, pathways involving transitions to microstate D were negatively correlated with WMH volume and positively correlated with MMSE score.

### 3.6 Classification prediction models

**FIGURE 5:**
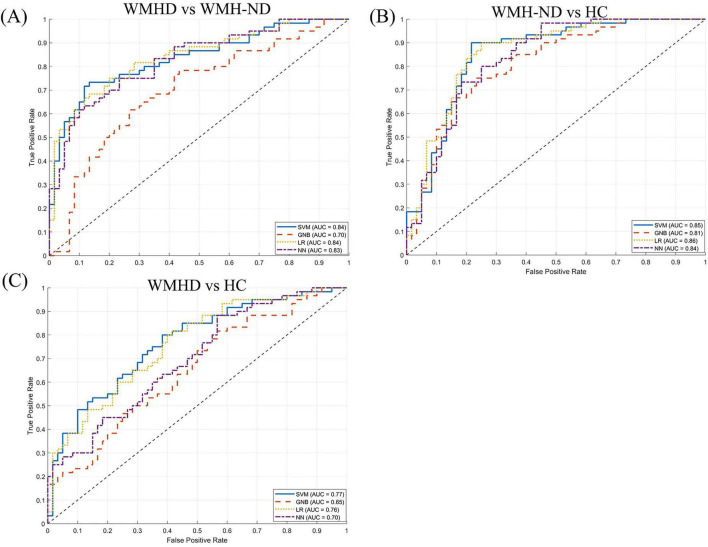
ROC curves of classification prediction models (SVM, GNB, LR, and NN). **(A)** Model for distinguishing WMHD from WMH-ND; **(B)** model for distinguishing WMH-ND from HC; **(C)** model for distinguishing WMHD from HC. AUC values are shown in the legend.

For the classification model distinguishing WMHD from WMH-ND, the SVM and LR algorithms performed best, both achieving an accuracy of 76.67%, with precisions of 78.56 and 77.59%, recalls of 73.33 and 75.00%, F1 scores of 0.76 for both, and AUC values of 0.84 for both (Table 4 and Figure 5A). The NN model ranked second in performance, while the GNB model showed the relatively lowest performance. We further evaluated the classification performance of the EEG microstate features for distinguishing WMH-ND from HC and WMHD from HC. For the WMH-ND vs. HC comparison, the LR model achieved the best performance with an accuracy of 79.17%, precision of 79.66%, recall of 78.33%, F1 score of 0.79, and AUC of 0.86, followed closely by the SVM model (accuracy: 80.00%, AUC: 0.85) (Table 4 and Figure 5B). These results indicate that EEG microstate features can effectively differentiate WMH-ND patients from healthy controls, suggesting that microstate alterations are already detectable in WMH patients without clinically overt dementia. For the WMHD vs. HC comparison, the SVM model achieved the best performance with an accuracy of 68.33%, precision of 69.64%, recall of 65.00%, F1 score of 0.67, and AUC of 0.77 (Table 4 and Figure 5C). The relatively lower performance compared to the WMH-ND vs. HC classification may reflect the greater heterogeneity of EEG microstate alterations in advanced WMH pathology, or the overlapping effects of multiple neurodegenerative processes in demented patients.

**TABLE 4:** Comparison of model performance.

Models	Algorithms	Accuracy	Precision	Recall	F1-score	AUC
WMHD vs. WMH-ND	SVM	76.67%	78.56%	73.33%	0.76	0.84
	GNB	67.50%	69.81%	61.67%	0.65	0.70
	LR	76.67%	77.59%	75.00%	0.76	0.84
	NN	75.00%	75.00%	75.00%	0.75	0.83
WMH-ND vs. HC	SVM	80.00%	79.03%	81.67%	0.80	0.85
	GNB	73.33%	76.92%	66.67%	0.71	0.81
	LR	79.17%	79.66%	78.33%	0.79	0.86
	NN	76.67%	78.57%	73.33%	0.76	0.84
WMHD vs. HC	SVM	68.33%	69.64%	65.00%	0.67	0.77
	GNB	60.83%	65.12%	46.67%	0.54	0.65
	LR	67.50%	68.42%	65.00%	0.67	0.76
	NN	61.67%	62.96%	56.67%	0.60	0.70

## 4 Discussion

In this study, the WMHD group showed decreased temporal parameters of microstate C and prolonged durations of microstates A and B. The WMH-ND group also showed decreased microstate C parameters, with compensatory increases in the mean duration and time coverage of microstate D. Transitions toward microstate C were significantly reduced in both patient groups, whereas transition probabilities involving microstate D were lower in the WMHD than in the WMH-ND group. Correlation analyses revealed that temporal parameters and transition probabilities toward microstates A and B were positively correlated with WMH volume and negatively correlated with MMSE score. Conversely, those of microstate D showed the opposite correlations. Among the classification models, SVM and LR achieved moderate diagnostic performance.

The microstate alterations observed in WMH patients are likely rooted in the underlying pathophysiology of chronic cerebral hypoperfusion and microvascular ischemia, which are hallmark features of aCSVD. Long penetrating arterioles supplying periventricular and deep white matter are particularly vulnerable to ischemic damage due to their limited collateral circulation and watershed distribution (Pantoni, 2010). Reduced cerebral blood flow in these vulnerable regions—particularly in white matter tracts such as the cingulum bundle, uncinate fasciculus, and extreme capsule—may impair axonal transmission and synaptic synchronization, manifesting as the reduced temporal parameters of microstate C observed in our study. Importantly, individuals with higher WMH loads exhibited decreased cerebral blood flow and reduced functional connectivity strength in regions encompassing the salience network (Huang et al., 2021), which is closely associated with microstate C. Moreover, corpus callosum perfusion was significantly lower in patients with severe versus mild WMH, and identified specific perfusion metrics as independent predictors of WMH progression (Yu et al., 2025). Collectively, these findings support the notion that region-specific hypoperfusion in strategic white matter pathways may preferentially disrupt the structural connectivity of large-scale brain networks, manifesting as the altered microstate dynamics observed in our WMH patients. The spatial distribution of WMH complements these findings. Although TWMH was identical between the two patient groups, the WMHD group showed a greater periventricular predominance. Periventricular tracts—including the cingulum bundle, superior longitudinal fasciculus, and corona radiata—subserve long-range cortico-cortical connections essential for executive and attentional functions. Greater involvement of these strategic fibers may disrupt large-scale network integration more profoundly than deep lesions of equivalent volume, potentially explaining discordant cognitive outcomes despite similar global WMH loads (Zhang et al., 2023; Qiao et al., 2022; Karvelas and Elahi, 2023). These perfusion-related mechanisms provide a plausible anatomical substrate linking white matter pathology to the suppression of salience network activity reflected by microstate C abnormalities.

One of the most compelling findings of this study is the differential behavior of microstate D between the two patient groups: WMH-ND patients showed increased microstate D parameters, whereas WMHD patients exhibited significant reductions. This pattern suggests a dynamic evolution of the attention network from early compensatory enhancement to late decompensated failure. We propose that the initial compensatory enhancement in WMH-ND reflects the recruitment of additional attentional network resources—likely through increased neural synchronization and engagement of alternative neural pathways—to maintain cognitive function in the face of accumulating white matter injury (Stern, 2009). This compensatory mechanism may be mediated by the superior longitudinal fasciculus and inferior fronto-occipital fasciculus, which are critical white matter tracts supporting attentional and executive functions (Schmahmann et al., 2008). The subsequent “decompensation” in the WMHD group may reflect the exhaustion of this neural reserve capacity as WMH burden and associated perfusion deficits exceed a critical threshold. Progressive perfusion decline in these attention-related white matter pathways could disrupt the functional integrity of the dorsal attention network, leading to the observed reduction in microstate D parameters. This compensation-decompensation transition may represent a tipping point beyond which cognitive function deteriorates irreversibly, highlighting the importance of early intervention in patients with WMH.

Our findings align with and extend previous EEG and microstate studies in CSVD and vascular cognitive impairment (VCI). Babiloni et al. reported that VCI patients showed reduced alpha rhythms and increased delta/theta rhythms, suggesting a global slowing of oscillatory activity (Babiloni et al., 2021). Torres-Simón et al. found that VCI patients exhibited slowed frequency patterns and disrupted inter- and intra-hemispheric connectivity, reflecting widespread functional network disturbances (Torres-Simon et al., 2022). More specifically, Jin et al. recently demonstrated that CSVD patients with cognitive impairment showed significantly lower appearance rates for all four canonical microstates (A–D) compared to healthy controls, along with prolonged durations of microstates B, C, and D, and a reduced transition probability from C to A (Jin et al., 2025). These authors also reported that the occurrence rate of microstate D was positively correlated with MMSE scores, while the A→B transition was negatively correlated with cognitive performance. Our findings extend these observations in several important ways. First, by including both demented (WMHD) and non-demented (WMH-ND) patient groups, we were able to delineate differential microstate patterns associated with the progression of cognitive decline. Second, we identified a compensation-decompensation transition in microstate D that has not been previously reported in CSVD. Third, the correlation and classification analyses in our study provide a more comprehensive characterization of the relationship between microstate alterations and cognitive function. Collectively, these findings reinforce the notion that microstate analysis can capture distinct stages of cognitive impairment in WMH patients, with potential implications for early diagnosis and disease monitoring.

The clinical and translational implications of our findings are noteworthy. The altered transition patterns observed in WMH patients—particularly the increased transitions between microstates A and B (sensory networks) and the reduced transitions toward microstate C (salience network)—likely reflect impaired “network switching” efficiency. This impairment may underlie several cognitive deficits commonly seen in CSVD, including executive dysfunction, attention lability, and processing speed slowing (Pantoni, 2010; Markus and de Leeuw, 2022). The transition probability metrics, especially those involving the salience and attention networks, may serve as functional correlates of cognitive reserve and as predictive biomarkers for conversion from non-demented to demented states. Furthermore, given that the observed microstate alterations appear to be linked to regional hypoperfusion, they may be potentially reversible with early vascular risk factor modification or revascularization strategies (Baezner et al., 2008). This raises the possibility that EEG microstate analysis could be used not only for early detection and risk stratification but also for monitoring treatment response in clinical trials targeting vascular cognitive impairment. The classification models developed in our study, with moderate diagnostic performance, further support the potential utility of EEG microstate features as non-invasive, cost-effective screening tools for WMH-related dementia in primary care or resource-limited settings.

EEG microstate analysis identifies quasi-stable scalp potential topographical patterns lasting tens to hundreds of milliseconds in EEG signals, thereby revealing functional network characteristics of the brain under different cognitive states. These microstate patterns are typically classified into four canonical classes (A, B, C, D), each associated with specific brain network activities based on extensive EEG-fMRI simultaneous recording studies (Britz et al., 2010). Using simultaneous EEG-fMRI, Britz et al. demonstrated that the four canonical microstates correspond to four distinct resting-state networks (RSNs), with the time course of each microstate significantly predicting BOLD signal fluctuations in spatially distinct networks. Subsequent source localization studies have further refined these associations: microstate A is spatially correlated with negative BOLD activation in bilateral superior and middle temporal gyri, corresponding to the auditory network; microstate B is associated with negative BOLD activation in bilateral extrastriate visual areas, corresponding to the visual network; microstate C is related to positive BOLD signals in the anterior cingulate cortex and bilateral inferior frontal gyri, consistent with the salience network; and microstate D is correlated with negative BOLD activation in ventral frontal and parietal areas, corresponding to the dorsal attention network. These functional assignments have been consistently validated across multiple independent studies and are now well-established in the field (Michel and Koenig, 2018; Khanna et al., 2015; Custo et al., 2017).

These microstate abnormalities stand in sharp contrast to the pathological features of Alzheimer’s disease (which predominantly exhibit microstate B/D abnormalities; Smailovic et al., 2019, Lin et al., 2021), indicating that the effects of white matter hyperintensities and amyloid deposition on neural networks differ. WMH may preferentially disrupt long-range connections of the salience network centered on the insula and cingulate cortex (Vipin et al., 2023). Alterations in microstate transition probabilities also revealed impaired switching efficiency of brain networks in WMH-related pathology. Both the WMHD and WMH-ND groups showed significant suppression of transition pathways toward microstate C. In WMHD group, transition probabilities between microstates A and B were significantly increased, and these transitions were positively correlated with WMH volume and negatively correlated with MMSE score. These findings suggest that white matter lesions cause neural activity to become more confined to lower-level sensory processing networks (microstates A and B), hindering the switch to higher-order salience networks (microstate C) and thereby impairing the integration efficiency of higher-order cognitive information (Michel and Koenig, 2018; Khanna et al., 2015).

Microstate analysis not only provides a perspective for revealing the neuroelectrophysiological alterations underlying cognitive impairment but also opens new avenues for early diagnosis. Wu et al. successfully distinguished patients with mild cognitive impairment (MCI) from healthy controls by integrating microstate features with machine learning algorithms, highlighting the diagnostic value of microstate characteristics in identifying early cognitive impairment (Wu et al., 2025). More importantly, a longitudinal study by Engedal et al. demonstrated that EEG-based microstate analysis can predict the risk of conversion from subjective cognitive decline and MCI to dementia (particularly Alzheimer’s disease), further supporting its important application value in early warning and prognostic assessment of the disease (Engedal et al., 2020).

In our classification analyses, we extended the evaluation to three pairwise comparisons using EEG microstate features. For distinguishing WMHD from WMH-ND, SVM, and LR achieved moderate performance with an AUC of 0.84. Notably, the best discriminative performance was observed for WMH-ND versus HC, with LR achieving an AUC of 0.86, indicating that EEG microstate alterations are already detectable in WMH patients without clinically overt dementia. In contrast, the classification between WMHD and HC showed relatively lower performance (SVM: AUC = 0.77), which may reflect the greater heterogeneity of EEG microstate alterations in advanced WMH pathology or the confounding effects of age-related comorbidities and concomitant neurodegenerative processes in demented patients. The gradient pattern of classification performance—AUC values decreasing from WMH-ND vs. HC (0.86) to WMHD vs. WMH-ND (0.84) to WMHD vs. HC (0.77)—is clinically plausible: microstate features are most discriminative when comparing WMH patients (even without dementia) to healthy controls, whereas distinguishing demented WMH patients from healthy controls is more challenging, possibly due to the overlapping effects of multiple pathological processes in advanced disease stages. These findings further support the potential utility of EEG microstate features as non-invasive biomarkers for early detection of WMH-related cognitive impairment, and the classification models developed in this study provide a foundation for future validation in larger, multicenter cohorts.

## 5 Limitations and future directions

Several limitations of this study should be acknowledged. First, the cross-sectional design precludes causal inferences. Future longitudinal studies with repeated EEG and neuropsychological assessments are needed to clarify the dynamic trajectory of microstate changes and their predictive value for conversion to dementia. Second, the WMH-ND group was broadly defined and may include both cognitively normal individuals and those with mild cognitive impairment, which could dilute observed differences. More refined cognitive subtyping in future studies would help address this issue. Third, while we integrated perfusion-related mechanisms into our interpretation, we did not directly measure cerebral blood flow. Future multimodal imaging studies (e.g., ASL, DTI, and EEG) are needed to confirm the structure-function-perfusion relationship. Fourth, the modest sample size from a single center limits generalizability and the robustness of classification models. Larger, multicenter prospective studies with external validation are essential. Fifth, we cannot entirely rule out co-morbid pathology such as covert Alzheimer’s disease. Incorporating cerebrospinal fluid or PET biomarkers in future studies would help disentangle vascular from neurodegenerative contributions. Finally, the 19-channel EEG system provides limited spatial resolution compared to high-density setups. Future studies using higher-density EEG may improve the precision of microstate analysis.

## 6 Conclusion

Patients with WMH exhibit significant alterations in EEG microstate temporal parameters and transition probabilities. These alterations are closely correlated with WMH burden and cognitive function, suggesting potential neuroelectrophysiological mechanisms underlying WMH-related cognitive impairment. Furthermore, classification prediction models based on EEG microstate features demonstrate potential for practical application and warrant validation in future studies.

## Data Availability

The raw data supporting the conclusions of this article will be made available by the authors, without undue reservation.

## Footnotes

1
www.statistical-modelling.de/lst.html
